# Health-related quality of life and migration: A cross-sectional study on elderly Iranians in Sweden

**DOI:** 10.1186/1477-7525-5-60

**Published:** 2007-11-23

**Authors:** Afsaneh Koochek, Ali Montazeri, Sven-Erik Johansson, Jan Sundquist

**Affiliations:** 1 Center for Family and Community Medicine, Karolinska Institute, Huddinge, Sweden; 2 Iranian Institute for Health Sciences Research, Tehran, Iran; 3 Stanford Prevention Research Center, Stanford University School of Medicine, California, USA

## Abstract

**Background:**

Although elderly Iranian immigrants in Sweden are the largest elderly group born outside Europe, little is known about their health-related quality of life (HRQL). The aim of this study was to examine the association between migration status and HRQL in a comparison of elderly Iranians in Iran, elderly Iranian immigrants in Sweden, and elderly Swedes in Sweden.

**Methods:**

The Short Form Health Survey (SF-36) was administered to a total of 625 men and women aged 60–84 years to collect HRQL information on elderly Iranians in Sweden (n = 176) and elderly Iranians in Iran (n = 298). A Swedish control group (n = 151) was also randomly selected from the general population. Multiple linear regression procedures were applied to analyze data while adjusting for age, which was categorized into 60–69, and 70–84 years, and education.

**Results:**

Iranian women in Sweden with shorter times of residence scored lower on vitality (β-coefficient = -7.9, 95% CI = -14.3 to -1.5) compared with other women in this study. The lower vitality dimension score remained nearly unchanged in the main model (β-coefficient = -7.3, 95% CI = -13.7 to -0.9). A longer period of residence in Sweden had a positive association with social functioning (β-coefficient = 14.1, 95% CI = 3.1–25.1) and role limitation due to emotional problems (β-coefficient = 18.3, 95% CI = 1.4–35.2) among elderly Iranian women. In general, the Swedish subsample scores higher on all dimensions of the SF-36 among women and in six out of eight among men in relation to the rest of the subsamples.

**Conclusion:**

The HRQL of elderly Iranians in Sweden was more like that of their countrymen in Iran than that of Swedes, who reported a better HRQL than Iranians in this study. However, length of time since migration to Sweden is not associated with poorer HRQL among elderly Iranians. The association varied, however, with sex. Elderly Iranian women showed an increase in two of eight dimensions of the SF-36 with additional years in Sweden, whereas, among elderly Iranian men, additional years in Sweden were not associated with HRQL.

## Background

Despite the fact that the elderly Iranian immigrants in Sweden are the largest elderly group born outside of Europe [1], research findings on their health-related quality of life (HRQL) are scarce. This is an important public health issue because the proportion of elderly immigrants in Sweden is increasing and cardiovascular disease (CVD) is one of the main causes of morbidity and mortality among older people [2,3]. Moreover, the increase in the prevalence of CVD in the elderly can lead to impairment of the HRQL [4]. This becomes even more important on considering that most of the immigrants in Sweden live longer than their fellow-countrymen in the native country [5] and a longer life expectancy prolongs the duration of exposure to risk factors and results in an elevated risk of CVD clinical events and also of other chronic disorders.

Recent studies on migration and health tend to focus on risk factors for CVD in younger generations [6-8] and have demonstrated that the length of time since migration is associated with a high prevalence of a disadvantageous risk factor profile. Moreover, a small number of studies have investigated the health of elderly immigrants in Sweden and shown that foreign-born elderly individuals report poorer health than native-born ones [9,10]. These findings raise the question of whether the observed health disadvantages among elderly immigrants reflect differences in the underlying health status of people from the country of origin, in the new country, or are related to the time of residence in the new country. We studied this question comparing two groups of older Iranian immigrants with shorter and longer times of residence in Sweden with similar, non-migrant Iranians in Iran and the general aging population in Sweden.

This study was aimed at bridging the gap in knowledge by examining whether there is an association between migration status, i.e. being an elderly Iranian in Iran, an elderly Iranian immigrant in Sweden, or an elderly Swede in Sweden, and the health-related quality of life. Additionally, we wished to examine whether the length of time since migration to Sweden is associated with the HRQL, taking into account the variables age and education.

## Methods

### Measure of health-related quality of life

We used the Short Form Health Survey (SF-36) to measure the quality of life. This is a well-known general instrument for measuring the health-related quality of life that is available both in Farsi (the Iranian language) and Swedish [11,12]. The SF-36 measures eight health-related concepts: physical functioning (PF-10 items), role limitations due to physical problems (RP-4 items), bodily pain (BP-2 items), general health perceptions (GH-5 items), vitality (VT-4 items), social functioning (SF-2 items), role limitations due to emotional problems (RE-3 items), and perceived mental health (MH-5 items). The first four dimensions are related to physical health, while the last four are related to mental health. The areas cover activities of daily living, emotional state, pain, fatigue, social participation, and perceptions of health. In addition, a single item that provides an indication of a perceived change in the general health status over a one-year period (health transition) is also included in the SF-36. The items can be summed up to give scores of 0–100. A higher score indicates a better HRQL for a particular area [12].

The Iranian version of SF-36, which had previously been translated into Farsi and validated [11], was administered to the Iranian group in this study. The Iranian version was produced in the International Quality of Life Assessment (IQOLA) Project to match the original SF-36 from the United States of America.

### The study population

All Iranian-born persons aged 60–84 who resided in the township of Kista, Stockholm (n = 286), were invited to participate in the study via a letter written in both Swedish and Farsi. One hundred and seventy-six persons (65%) agreed to participate in the study. Interviews were conducted face-to-face in the participants' native language, Farsi, using a questionnaire based on material produced for the Swedish Annual Level of Living Survey by Statistic Sweden.

The non-response analysis of the Iranian group in Sweden was conducted by telephone. In total, 16 women and 5 men were contacted in this way. There were no significant differences between the respondents and non-respondents with regard to sex, education, and self-reported health. However, the non-respondents were slightly older (p < 0.05) (mean age ± SD = 72.8 ± 7.7 years) than the participants (mean age ± SD = 70.5 ± 7.0 years).

#### Power

The original sample size was calculated to detect a minimum differences of 6.5 (SD = 21) units in general health (SF-36) between elderly Iranian immigrants in Sweden and non-migrant groups in Iran and Sweden with an α = 0.05 and a statistical power of 80%. In addition, we also anticipated a 40% non-response rate for the Iranian-born group in Sweden and determined that the recruitment goal would be 175 participants in the Iranian immigrant group. The Swedish-born group was matched for age and sex and the Iranian group in Iran was matched for age.

The Iranian group in Iran consisted of 298 randomly sampled healthy Iranian-born persons aged 60–84, living in 22 urban districts in Tehran, who were selected using a stratified multi-stage area sampling procedure. The response rate in this sample was 87%. A team of trained interviewers collected data and all participants were interviewed in their home [11].

The SF-36 score of each of the Iranian groups in Iran and Sweden was matched with the Swedish population norms for sex and age (n = 151), which were randomly drawn from the Swedish SF-36 national normative database (n = 8930) [12].

### Independent variables

*Age *was categorized into the following age groups: 60–69 and 70–84.

*Migration status *was defined as Iranians in Iran, Iranians in Sweden, or Swedes. In addition, the Iranian group in Sweden was further divided according to time of residence in Sweden, which was based on the self-reported calendar year when the Iranian participant immigrated to Sweden. Thus, the Iranians were categorized as either "immigrated in 1988 or earlier" or "immigrated in 1989 or later". This categorization was performed since previous research has demonstrated an association between time in Sweden and risk factors for CVD [7]. The cut-off point, 1988–89, was chosen in order to obtain two groups of uniform size.

*Education *was used as an indicator of socioeconomic status and the participants were classified into two categories: (1) ≤ 9 years of education and (2) > 9 years of education.

### Statistical analysis

Scores for the eight dimensions were coded, summed up, and ranked on a scale from 0 (worst possible health) to 100 (best possible health) by means of the SAS software package, using the method described in the user manual [12] and are reported for the two sexes and for each of the eight dimensions. The SF-36 scores (continuous) were analyzed by multiple linear regressions using STAT software [13]. The following reference categories were chosen: Iranians in Iran (migration status), 60–69 years (age), and ≤9 years (education). By definition, the reference group has a β-estimate of zero so that the value of the β-coefficient corresponds to the difference in scores for each dimension and category compared to the reference category. The results are shown as β-coefficients with 95% confidence intervals (CIs).

If repeated random samples are drawn and a 95% confidence interval for the estimated parameter is constructed for each sample, 95% of all intervals will contain the unknown (true) parameter μ. In our study we estimated β-coefficients, which indicate that if the CI contains zero, the β-coefficients are non-significant.

Two models were taken into consideration: the first one was unadjusted (crude model) and the second one (main model) was adjusted for age and education.

### Ethical considerations

This study was approved by the Karolinska Institute's Ethics Committee, Reg. No. 92/03, March 10, 2003. All participants in the Iranian group in Sweden gave their informed consent to participate in the study. There was no risk of identification of the participants in this study because names and personal identification numbers were deleted before the analysis started. All personal registration numbers have been replaced by serial numbers. The use of the research database is restricted on the conditions of the highest security. Only the main author has access to the data. All data will be presented as group data without any possibility of identifying individuals.

## Results

The characteristics of the study sample by migration status and sex are shown in Table 1. The numbers of women were higher among Iranians in Sweden and Swedes (120 and 102, respectively) than those of men (56 and 49, respectively) from the same groups and they were slightly older than the men. Iranians who immigrated to Sweden in 1988 or earlier were more educated than other groups in this study, whereas Iranian women in Iran were less educated (90%).

**Table 1:** Characteristics of the study sample (values are numbers, with percentages given in parentheses)

		Migration status							
		
		Iranians in Iran		Iranians in Sweden				Swedes	
				Immigrated in 1989 or later		Immigrated in 1988 or before			

Variable	Level	Men n = 147	Women n = 151	Men n = 27	Women n = 70	Men n = 29	Women n = 50	Men n = 49	Women n = 102
Age	60 – 69 years	79 (54%)	93 (62%)	16 (60%)	34 (49%)	13 (45%)	24 (48%)	30 (61%)	57 (56%)
	70 – 84 years	68 (46%)	58 (38%)	11 (40%)	36 (51%)	16 (55%)	26 (52%)	19 (39%)	45 (44%)
Mean age ± SD (years)		68.9 ± 6.6	67.0 ± 5.9	69.3 ± 5.2	70.2 ± 7.2	70.2 ± 6.9	70.8 ± 7.7	67.9 ± 6.0	68.6 ± 6.9
Education	> 9 years	48 (33%)	15 (10%)	18 (66%)	13 (19%)	21 (72%)	19 (38%)	11 (22%)	24 (24%)
	≤ 9 years	99 (67%)	136 (90%)	9 (33%)	57 (81%)	8 (28%)	31 (62%)	38 (78%)	75 (76%)

**Figure 1:**
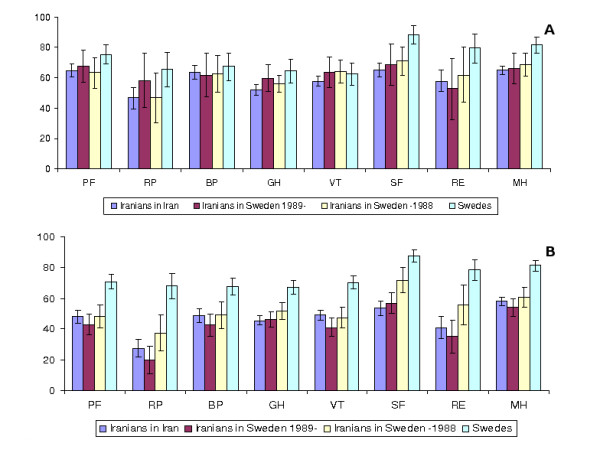
**A: **Unadjusted mean scores and confidence interval for SF-36 dimension scores by migration status for men. **B: **Unadjusted mean scores and confidence interval for SF-36 dimension scores by migration status for women.

The mean scores and standard deviation of the SF-36 dimension scores by migration status are presented in Figures 1a and 1b for men and women, respectively.

The findings in the crude model indicate that Iranian women in Sweden with a shorter time of residence scored a lower HRQL than Iranian women in Iran on six of eight dimensions of the SF-36. However, only the score for vitality was significantly lower (β-coefficient = -7.9, 95% CI = -14.3–1.5) and remained nearly unchanged after adding the effect of age and education in the main model (β-coefficient = -7.3, 95% CI = -13.7–0.9) (Table 2). Furthermore, a longer time of residence in Sweden was more likely to have a positive effect on all of the SF-36 subscales among elderly Iranian women. Additional analysis showed that these positive effects were statistically significant for social functioning (β-coefficient = 14.1, 95% CI = 3.1–25.1) and role limitation due to emotional problems (β-coefficient = 18.3, 95% CI = 1.4–35.2) and just slightly significant for role limitations due to physical problems (β-coefficient = 14.6, 95% CI= -0.4–29.6) after adjusting for age and education (data are not shown but are available from the corresponding author).

**Table 2:** β-coefficient and 95% confidence interval (CI) for SF-36 dimensions in two models

**Variable**	**Crude model**		**Main model (adjusted for age and education)**	
	
	Women	Men	Women	Men
PF				
Iranians in Iran	0 (Reference)	0 (Reference)	0 (Reference)	0 (Reference)
Iranians in Sweden 1989-	-5.1 (-12.7 – 2.6)	2.8 (-7.8 – 13.5)	-3.3(-10.7 – 4.1)	-0.5(-11.2 – 10.1)
Iranians in Sweden -1988	0.2 (-8.4 – 8.9)	-1.4 (-11.5 – 8.8)	1.4 (-7.3 – 10.0)	-4.4 (-14.5 – 5.6)
Swedes	**22.7 (15.7 – 29.6)**	**10.7 (2.2 – 19.2)**	**23.6 (16.8 – 30.4)**	**10.6 (2.4 – 18.8)**
RP				
Iranians in Iran	0 (Reference)	0 (Reference)	0 (Reference)	0 (Reference)
Iranians in Sweden 1989-	-7.8 (-18.9 – 3.6)	11.7 (-6.0 – 29.4)	-7.5 (-18.7 – 3.6)	2.3 (-15.3 – 19.9)
Iranians in Sweden -1988	9.9 (-2.7 – 22.4)	0 (-17 – 16.9)	7.9 (-5.2 – 20.9)	-7.3 (-23.6 – 9.3)
Swedes	**40.4 (30.2 – 50.5)**	**19.0 (4.8 – 33.1)**	**40.9 (30.7 – 51.2)**	**19.0 (5.4 – 32.5)**
BP				
Iranians in Iran	0 (Reference)	0 (Reference)	0 (Reference)	0 (Reference)
Iranians in Sweden 1989-	-6.3 (-14.6 – 1.9)	-1.9 (-14.8 – 11.1)	-7.1 (-15.5 – 1.2)	-6.0 (-19.2 – 7.2)
Iranians in Sweden -1988	0.16 (-9.2 – 9.5)	-1.1 (-13.3 – 11.0)	-1.8 (-11.6 – 7.9)	-3.6 (-15.9 – 8.7)
Swedes	**18.9 (11.5 – 26.3)**	3.8 (-6.1 – 13.7)	**18.8 (1.3 – 26.3)**	3.6 (-6.0 – 13.3)
GH				
Iranians in Iran	0 (Reference)	0 (Reference)	0 (Reference)	0 (Reference)
Iranians in Sweden 1989-	0.8 (-5.2 – 6.7)	7.7 (-1.6 – 17.0)	0.6 (-5.4 – 6.6)	5.3 (-4.2 – 14.8)
Iranians in Sweden -1988	6.2 (-0.5 – 12.8)	4.1 (-4.6 – 12.8)	5.0 (-2.0 – 12.3)	1.7 (-7.2 – 10.6)
Swedes	**21.6 (16.2 – 27.0)**	**12.4 (4.8 – 20.0)**	**21.4 (16.0 – 26.9)**	**13.1 (5.6 – 20.5)**
VT				
Iranians in Iran	0 (Reference)	0 (Reference)	0 (Reference)	0 (Reference)
Iranians in Sweden 1989-	**-7.9 (-14.3 – -1.5)**	5.9 (-3.1 – 14.8)	**-7.3 (-13.7 – -0.9)**	3.6 (-5.6 – 12.8)
Iranians in Sweden -1988	-1.3 (-8.5 – 5.9)	6.4 (-2.0 – 14.8)	-1.6 (-9.0 – 5.9)	6.5 (-2.0 – 15.1)
Swedes	**21.2 (15.4 – 27.0)**	5.0 (-2.0 – 12.1)	**21.2 (15.4 – 27.0)**	3.9 (-3.0 – 10.9)
SF				
Iranians in Iran	0 (Reference)	0 (Reference)	0 (Reference)	0 (Reference)
Iranians in Sweden 1989-	3.7 (-4.1 – 11.5)	3.6 (-7.6 – 14.9)	4.0 (-4.0 – 12.0)	2.1 (-9.6 – 13.8)
Iranians in Sweden -1988	**18.3 (9.5 – 27.1)**	6.2 (-4.3 – 16.8)	**18.6 (9.3 – 27.9)**	4.8 (-6.2 – 15.7)
Swedes	**34.1 (27.2 – 41.1)**	**23.6 (15.0 – 32.2)**	**35.2 (28.0 – 42.3)**	**23.7 (15.1 – 32.3)**
RE				
Iranians in Iran	0 (Reference)	0 (Reference)	0 (Reference)	0 (Reference)
Iranians in Sweden 1989-	-5.8 (-18.1 – 6.4)	-5.2 (-23.3 – 13.0)	-8.3 (-20.9 – 3.7)	-8.0 (-27.0 – 10.9)
Iranians in Sweden -1988	**14.5 (0.5 – 28.5)**	4.1 (-13.2 – 21.4)	9.7 (-4.9 – 24.2)	1.7 (-16.1 – 19.6)
Swedes	**37.5 (26.3 – 48.7)**	**21.7 (7.3 – 36.1)**	**37.2 (25.8 – 48.6)**	**21.7 (7.2 – 36.1)**
MH				
Iranians in Iran	0 (Reference)	0 (Reference)	0 (Reference)	0 (Reference)
Iranians in Sweden 1989-	-3.9 (-9.6 – 1.9)	1.2 (-6.8 – 9.1)	-4.6 (-10.5 – 1.2)	0.8 (-7.5 – 9.1)
Iranians in Sweden -1988	2.5 (-4.0 – 8.9)	3.8 (-3.8 – 11.4)	1.0 (-5.8 – 7.8)	4.1 (-3.7 – 11.9)
Swedes	**23.4 (18.2 – 28.6)**	**16.7 (10.2 – 23.1)**	**22.8 (17.5 – 28.2)**	**16.5 (10.0 – 22.9)**

PF = physical functioning, RP = role limitations due to physical problems, BP = bodily pain, GH = general health perceptions, VT = vitality, SF = social functioning, RE = role limitations due to emotional problems, MH = perceived mental health

The dimensions assessing role limitations due to physical problems, general health perceptions, social functioning, role limitations due to emotional problems, and mental health showed better outcomes for Iranian women with a longer time of residence compared with Iranian women in Iran. However, only the β-coefficients for social functioning and role limitations due to emotional problems were significantly higher in the crude model. The β-coefficient for social functioning remained significantly higher even after adjusting for age and education in the main model (Table 2). Swedish women in this study score higher on all dimensions of the SF-36 in relation to Iranian women. Swedish men scored higher for physical function, role limitations due to physical problems, general health perceptions, social functioning, role limitations due to emotional problems, and perceived mental health in relation to Iranian men.

## Discussion

The main finding of this study was that, in general, the HRQL of elderly Iranians in Sweden did not decrease with the time of residence according to dimensions of the SF-36. However, the results of examining whether the length of time since migration to Sweden is associated with HRQL showed few significant differences. Another finding was that the mean scores of HRQL for both Iranian groups were lower than those of the Swedish general population in all dimensions among women and in six of eight dimensions among men. Moreover, the finding indicated that the association between the length of time since migration to Sweden and HRQL varied with sex. Elderly Iranian women with a shorter time of residence in Sweden reported a lower vitality than Iranian women in Iran. Nevertheless, the social functioning and role limitation due to emotional problems increased with additional years in the new country. In contrast, among elderly Iranian men, additional years in Sweden were not associated with HRQL.

Our observation that the elderly Iranian immigrants reported poorer health than Swedes agreed with other studies which have confirmed that foreign-born elderly individuals report poorer health than native-born elderly individuals [9,10]. The finding that elderly Iranian women with a shorter time of residence in Sweden had an impaired vitality compared to Iranian women in Iran agreed in part with Bentham's theory [14], which identified poor health as a reason for migration to a new country among elderly people in order to be closer to their families. Meanwhile, the fact that this group of immigrant women is more likely not to experience a poorer HRQL with additional years in Sweden is not in accord with Findley's study [15], which claims that elderly persons with poor health will be more likely to experience additional impairment of their health after migration.

### Possible pathways

In this study, the HRQL of elderly Iranians in Sweden was more like that of their countrymen in Iran than that of Swedes. For this reason, we argue that the observed lower HRQL among elderly Iranian immigrants, compared to elderly Swedes, reflects the underlying status of HRQL among people from the country of origin. On the other hand, the finding that length of time since migration to Sweden has no negative effect on health may possibly be due to a quite new migration pattern observed in elderly immigrants in Sweden, which is characterized by travelling between Sweden and their country of origin, spending long periods of time in each country. This new migration pattern has enabled elderly Iranians to succeed in taking advantage of the best of their original culture and the host country's culture [16].

The higher HRQL among elderly Swedes in this study may be due to potential cross-cultural differences in the perception of health, i.e. differences in perceived health or disease prevalence. In a population-based cross-sectional study from the 2001 California Health Interview Survey, it was estimated that differences in self-reported overall health between different ethnic groups may be due to different perceptions of health that are rooted in culture and language [17]. Moreover, individuals living in different cultural environments with the same disease may perceive their disease differently, which might affect the quality of life in a different way [18]. However, the extent to which cultural differences between elderly Iranians and Swedes influence the reported HRQL is not clear.

The finding of low vitality among Iranian women with a shorter period of residence in Sweden is alarming and might reflect the multiple health problems and high prevalence of CVD risk factors among Iranian women in Iran, which has been documented in many studies [19-25]. However, according to Bentham's theory [14], poor vitality due to a high prevalence of chronic diseases, such as CVD, might have been a reason for these women to migrate to Sweden.

### Method discussion

Socioeconomic status can be measured in different ways, although it is not possible to measure its full dimensions. In the current study, education was used only as a crude proxy of socioeconomic status. At first, we considered using income to characterize socioeconomic status. However, since 76% of the participants arrived in Sweden when they were 50 years of age or older they were not eligible for a full pension and therefore have very low incomes. Because of their limited pension rights and dependency on welfare aid, income is a blunt tool to differentiate individuals by socioeconomic status.

Even using occupation as an indicator of socioeconomic status seems to be less valid in this group of immigrants because nearly all of them are at the age of retirement. In addition, many immigrants in Sweden work in low-status jobs even though they have university degrees. Therefore, education was considered to be a more stable indicator of socioeconomic status in this particular group. Furthermore, education as a measure of socioeconomic status remains fairly unaffected over the course of life and the health status. In addition, health status may influence income and occupation, but not educational status.

One might argue, however, that a sample from Tehran is not necessarily representative of the entire country. In general, this is true, but since Tehran has became a multicultural metropolitan area it has been suggested that a sample from the general population in Tehran could at least be regarded as a representative sample of an urban population in Iran [11]. Regarding the Iranian sample in Stockholm, we studied a population-based representative sample of elderly Iranians in Kista/Stockholm and expect that the result would be generalizable to similar groups residing in other counties in Sweden. However, this expectation remains to be tested.

### Limitations and strengths

Some important limitations must be considered when interpreting the results of this study. First, given the cross-sectional nature of the results, the interpretation of the impact of migration on the HRQL is restricted. Future research with a longitudinal approach would be valuable in the area of migrant studies, but very difficult to perform.

The second limitation arises from the fact that we did not control for potentially confounding factors based on objective health status measures, e.g. weight, height, waist measurements, blood pressure, smoking, etc., in our analysis. These measurements are important to consider because of the accumulative prevalence of CVD risk factors in migrant and Iranian populations, particularly in Iranian women. However, objective health status measurements were not available in the data.

Finally, there is a lack of important variables such as social, ethnic, and cultural contexts in either the Stockholm sample or the Iranian sample. Although no previous study has documented an association between ethnicity, culture, and quality of life in Iranian people, we believe that the lack of an analysis of these variables in the results is an important limitation.

Despite the limitations, the present study has some strengths. Although the number of elderly Iranians in Sweden in the study is small, we had sufficient (80%) statistical power to detect medium-sized effects. Moreover, the well-defined control group, which constitutes a random sample of the Swedish population, is also a strength.

Although investigating differences in the HRQL of the two sexes was not one of the aims of this study, it is an important topic that future research can focus on. However, strategies and policies should include a special focus on recently arrived elderly female immigrants who showed a lower HRQL in some of the dimensions, compared to the other elderly Iranian immigrant women and to elderly Iranian women in Iran.

## Conclusion

In conclusion, this study suggests that length of time since migration to Sweden is not associated with a poorer health-related quality of life among elderly Iranians; however, the effect varied with sex. Elderly Iranian women showed an increase in two dimensions of the SF-36 with additional years in Sweden, whereas, among elderly Iranian men, additional years in Sweden were not associated with HRQL.

The Swedish general population reported a better HRQL than Iranians in this study, which may be due to potential cross-cultural differences in the perception of health, i.e., differences in perceived health or disease prevalence. Our results have practical implications for the health of elderly immigrants and particularly recently arrived elderly immigrant women.

## Abbreviations

PF: Physical functioning;

RP: Role limitations due to physical problems; 

BP: Bodily pain; 

GH: General health perceptions; 

VT: Vitality; 

SF: Social functioning; 

RE: Role limitations due to emotional problems; 

MH: Perceived mental health.

## Competing interests

The author(s) declare that they have no competing interests.

## Authors' contributions

AK is the corresponding author of the manuscript. She contributed as a principal researcher and writer, including drafting of the article and the analysis and interpretation of the data.

AM contributed material on the Iranian population in Tehran. He has made substantial contributions to the interpretation of the data.

SEJ participated in the design of the study and performed the statistical analysis.

JS made contributions to the design, acquisition, and interpretation of the data and participated in the writing process by commenting on the manuscript. He gave final approval of the version to be published

All authors read and approved the final manuscript.
